# Inhibition of Human Sulfotransferases by Phthalate Monoesters

**DOI:** 10.3389/fendo.2022.868105

**Published:** 2022-04-22

**Authors:** Hui Huang, Bei-Di Lan, Yu-Jing Zhang, Xiao-Juan Fan, Min-Cui Hu, Guo-Qiang Qin, Fei-Ge Wang, Yue Wu, Tao Zheng, Jun-Hui Liu

**Affiliations:** ^1^ Department of Cardiology, General Hospital of Ningxia Medical University, Yinchuan, China; ^2^ Department of CardioMetabolic Center, The First Affiliated Hospital of Xi’an Jiaotong University, Xi’an, China; ^3^ Tianjin Life Science Research Center, Department of Microbiology, School of Basic Medical Sciences, Tianjin Medical University, Tianjin, China; ^4^ Human Resources Department, The First Affiliated Hospital of Jinzhou Medical University, Jinzhou, China; ^5^ Department of Clinical Laboratory, The First Affiliated Hospital of Xi’an Jiaotong University, Xi’an, China

**Keywords:** phthalate esters (PAEs), sulfotransferases (SULTs), enzyme inhibition, *in silico* docking, *in vitro*–*in vivo* extrapolation

## Abstract

**Objective:**

This study aimed to investigate the inhibition of human important phase II metabolic enzyme sulfotransferases (SULTs) by phthalate monoesters, which are important metabolites of phthalate esters (PAEs).

**Method:**

Recombinant SULT-catalyzed metabolism of p-nitrophenol (PNP) was employed as the probe reactions of SULTs to investigate the inhibition of 8 kinds of phthalate monoesters towards SULT isoforms. An *in vitro* incubation system was utilized for preliminary screening, and 100 μM of phthalate monoesters was used. Inhibition kinetics were carried out to determine the inhibition of SULTs by phthalate monoesters.

**Result:**

Multiple phthalate monoesters have been demonstrated to exert strong inhibition potential towards SULT1A1, SULT1B1, and SULT1E1, and no significant inhibition of phthalate monoesters towards SULT1A3 was found. The activity of SULT1A1 was strongly inhibited by mono-hexyl phthalate (MHP), mono-octyl phthalate (MOP), mono-benzyl phthalate (MBZP), and mono-ethylhexyl phthalate (MEHP). Monobutyl phthalate (MBP), MHP, MOP, mono-cyclohexyl phthalate (MCHP), and MEHP significantly inhibited the activity of SULT1B1. MHP, MOP, and MEHP significantly inhibited the activity of SULT1E1. MOP was chosen as the representative phthalate monoester to determine the inhibition kinetic parameters (*K*_i_) towards SULT1B1 and SULT1E1. The inhibition kinetic parameters (*K*_i_) were calculated to be 2.23 μM for MOP-SULT1B1 and 5.54 μM for MOP-SULT1E1. *In silico* docking method was utilized to understand the inhibition mechanism of SULT1B1 by phthalate monoesters.

**Conclusions:**

All these information will be beneficial for understanding the risk of phthalate monoester exposure from a new perspective.

## Introduction

Phthalate esters (PAEs) are softening chemicals that are widely used in homes and industries as plasticizers (1). PAEs are commonly used and can easily cause harm to human body. Humans are exposed to PAEs mainly through respiratory inhalation, skin absorption, and dietary intake (2). PAEs have been found to affect the reproductive system in animals, and epidemiological studies have shown that high doses of PAEs affect the endocrine and reproductive systems in humans (3, 4). PAEs are eliminated through a two-step metabolic process in the body, and the metabolic processes contain phase I and phase II (conjugation) metabolism (5). PAEs are metabolized into phthalate monoesters in the human body through the phase I biotransformation (6). PAEs with low molecular weight are excreted in urine and feces mainly as monoesters; in contrast, phthalates with high molecular weight can be further metabolized by hydroxylation or oxidation after conversion to monoesters to produce a large number of oxidative metabolites (7). In recent years, more and more attention has been paid to the effect of phthalates on human health. Some studies have shown that phthalate monoesters have higher biological activity and toxicity than the parent compound (8).

Sulfotransferases (SULTs) are an enzyme that catalyzes the sulfuration of various endogenous and exogenous substrates. Sulfuryl transfer reaction has important basic biological significance (9). SULT catalyzes the transfer of a sulfuryl group provided by 3′-phosphoadenosine-5′-phosphosulfate (PAPS) to a receptor substrate, a process originally referred to as sulfate (10). Sulfation is an important conjugation pathway responsible for detoxification and elimination of a series of exogenous and endogenous small molecules in the body (11), and is an important mechanism for regulating the biological activity of various hormones and neurotransmitters (12). For example, SULT1C4 has been shown to sulfonate estrogen compounds, and sulfation is also an important pathway for thyroid hormone metabolism (13, 14). As a phase II metabolic enzyme, human SULTs show complex patterns of broad, differential, and overlapping substrate selectivity (15, 16). Although substrates of each subtype of SULTs have certain intersections, there are corresponding specific substrates. Thirteen expressed SULT genes are included in the human genome, and they encode 13 known SULT enzymes, although each gene does not produce a single protein (9). Many endogenous and exogenous chemicals are metabolized primarily in the liver; human SULTs include 4 families in which SULT1 and SULT2 enzymes are expressed in human liver (17).

The purpose of this study was to investigate the inhibitory effect of phthalate monoesters on the activities of human SULTs. Eight phthalate monoesters commonly used in industry were selected as inhibitors, and four SULT isoforms (SULT1A1, 1A3, 1B1, and 1E1) were selected for the experiments. In addition, preliminary inhibition screening, inhibition kinetic type, and parameters were determined, and *in silico* docking and *in vitro*–*in vivo* extrapolation (*IVIVE*) were carried out.

## Materials and Methods

### Chemicals and Reagents

**Figure 1:**
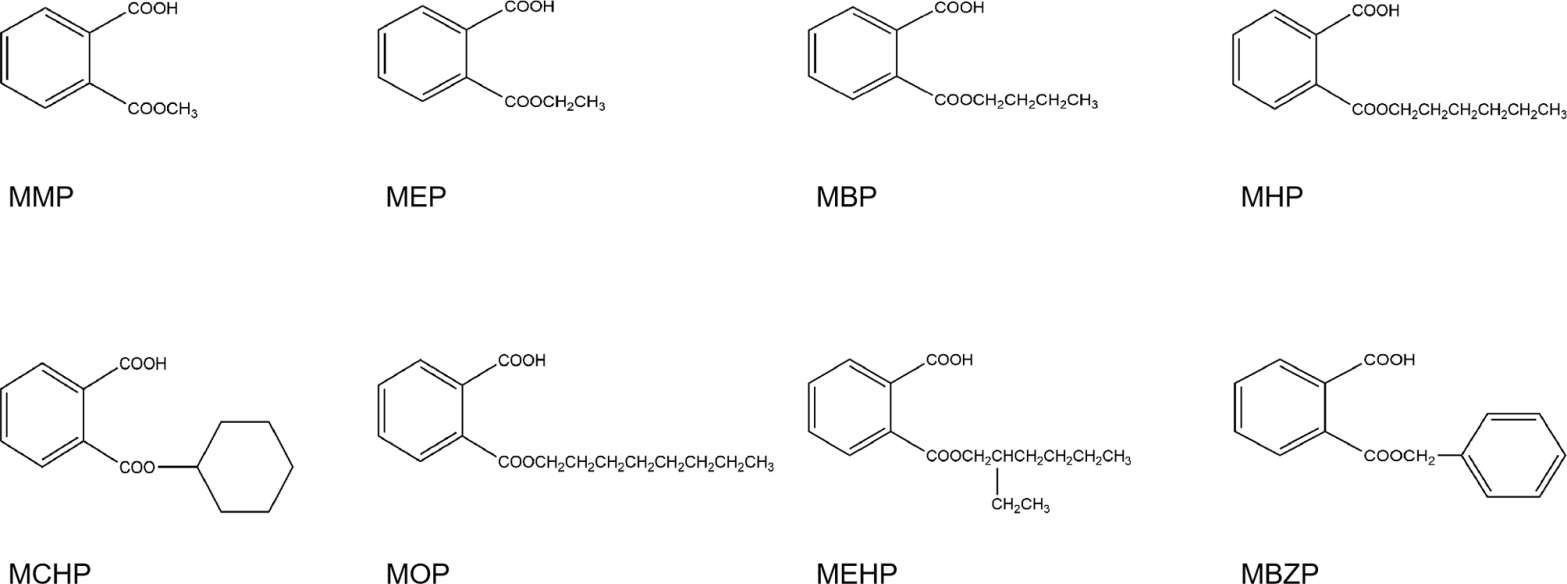
The structure of eight phthalate monoesters.

Eight phthalate monoesters (the structures of phthalate monoesters are given in **Figure 1**) were purchased from J&K Chemical (Beijing, China). Human SULT isoforms (SULT1A1, 1A3, 1B1, and 1E1) were obtained from BD Gentest Corp (Woburn, MA, USA). 4-Nitrophenol (PNP) and its sulfate PNP-S,3′-phosphoadenosine-5′-phosphosulfate (PAPS), Tris-HCl, and MgCl_2_ were purchased from Sigma-Aldrich (St. Louis, MO, USA). Ultra-performance liquid chromatography (UPLC) grade acetonitrile was purchased from Tianjin Saifurui Technology Ltd. Millipore Elix 5 UV and Milli-Q Gradient Ultra-Pure Water System were used for the preparation of ultra-pure water. Other reagents were of ultra-performance liquid chromatography (UPLC) grade or of the highest grade commercially available.

### Enzyme Activity Assays and Kinetic Study

The incubation system (total volume = 200 μl) containing 100 mM of Tris-HCl buffer (pH 7.4), 5 mM of MgCl_2_, 40 μM of PAPS, SULTs, and PNP. The concentrations of SULT 1A1, SULT1A3, SULT1B1, and SULT1E1 were 10 μg/ml, 10 μg/ml, 20 μg/ml, and 10 μg/ml, respectively. In the incubation process, a 3-minute pre-incubation was performed, and then PAPS was added to initiate the metabolic reaction. The reaction temperature was 37°C and the reaction time was 30–60 min. At the end of the reaction, 200 μl of ice-cold acetonitrile was added for termination. An incubation solution without phthalate monoesters was used as a control. Then, after centrifugation at 12,000 rpm, 10 μl of supernatant was taken for analysis with liquid chromatography (UPLC)-UV instrument. The column used for UPLC separation is a C18 column (4.6*200 mm, 5 μm, Kromasil) with a flow rate of 0.4 ml/min and a column temperature of 25°C. The mobile phase is 0.5% formic acid aqueous solution for phase A and acetonitrile for phase B. The gradient condition was used as follows: 0–6 min, 5% B; 6–8 min, 95% B; 8–14 min, 95% B; 14–17 min, 5% B. The detection wavelength was 280 nm. The incubation system (total volume = 200 μl) containing 100 μM of phthalate monoesters, Tris-HCl buffer (50 mM, pH 7.4), MgCl_2_ (5 mM), PAPS (40 μM), SULTs, and PNP was used to determine the Michaelis constant (*K*_m_) of PNP metabolites. The used concentration range was 1–400 μM for SULT1A1, 0.1–7 mM for SULT1A3, 0.1–1 mM for SULT1B1, and 0.1–1.2 mM for SULT1E1. The kinetic parameters (*V*_max_ and *K*_m_) were determined through fitting the data to the Michaelis–Menten equation or the substrate inhibition equation using Prism 4 software.

### Preliminary Investigation of the Inhibition of Phthalate Monoesters on SULTs

4-Nitrophenol was employed as a nonselective probe substrate for human SULT isoforms to investigate the inhibition of phthalate monoesters on SULT isoforms. The incubation system (total volume = 200 μl) containing 100 μM of phthalate monoesters,100 mM of Tris-HCl buffer (pH 7.4), 5 mM of MgCl_2_, and 40 μM of PAPS, and the concentrations of 4-nitrophenol and SULTs are different. The concentrations of 4-nitrophenol were 40, 1,200, 40, and 120 μM for SULT1A1, SULT1A3, SULT1B1, and SULT1E1, respectively. In addition, the concentrations of SULT isoforms were 10, 10, 20, and 10 μg/ml for SULT1A1, SULT1A3, SULT1B1, and SULT1E1, respectively. The absence of phthalate monoesters was set as the negative control. After pre-incubation at 37°C for 3 min, 40 μM PAPS were added for the reaction. The metabolic reaction is terminated by the addition of acetonitrile of the same volume and ensures that the incubation time can reach 30–60 min according to different SULTs. The optimized microsome protein concentration and incubation time were used to ensure linear velocity (*v*). The final mixture was centrifuged at 12,000 rpm for 10 min, and the supernatant of 10 μl was then used for ultra-performance liquid chromatography (UPLC)-UV instrument. All experiments were repeated in two independent experiments. Screening of phthalate monoesters was carried out with a SULT inhibition rate greater than 80% for subsequent experiments.

### Determination of Half Inhibition Concentration and Evaluation of Inhibition Kinetics

The concentration-dependent inhibition effect of phthalate monoesters on SULTs was investigated using different concentrations of phthalate monoesters (ranging from 0 μM to 100 μM), and the half inhibition concentration (IC_50_) was calculated. Based on different SULT isoforms, multiple concentrations of 4-Nitrophenol (covering the *K*_m_ value) and phthalate monoesters (covering the IC_50_ values) were used to determine the inhibition kinetics. Lineweaver–Burk plots are used to determine the type of inhibition kinetics. The second plot was used to calculate the inhibition kinetic parameters (*K*_i_), using the linear slope of the Lineweaver–Burk double reciprocal plot and the concentration of the inhibitor phthalate monoesters.

### *In Vitro*–*In Vivo* Extrapolation

*In vivo* inhibition magnitude of SULTs was determined through *IVIVE*. The following equation was used:


$$AUC_{i/}AUC=1+\left[I\right]/K_{i}$$

The terms are defined as follows: AUC_i_/AUC was the predicted ratio of *in vivo* exposure of xenobiotics or endogenous substances with or without the co-exposure of phthalate monoesters. [I] was the *in vivo* exposure concentration of phthalate monoesters, and the *K*_i_ was the *in vitro* inhibition constant. The standard used was as follows: [I]/*K*_i_ < 0.1, low possibility; 0.1 < [I]/*K*_i_ < 1, medium possibility; [I]/*K*_i_ > 1, high possibility.

### *In Silico* Docking

In order to better clarify the molecular interaction between phthalate monoesters and SULTs, an *in silico* docking method was used to dock the chemical structure of phthalate monoesters into the activity cavity of SULTs. Three-dimensional (3D) structure of SULT isoforms was established using a homology modeling method. Autodock Version 4.2 was employed to dock the flexible small molecule of phthalate monoesters into the rigid protein of SULTs. The non-polar hydrogen atoms of SULT enzymes were merged. The gridbox was generated with 60 × 60 × 60 in *X*, *Y*, and *Z* coordinates, covering the entire ligand-binding site. Lamarckian Genetic Algorithm (LGA) method was selected to possess molecular docking study for the binding of phthalate monoesters towards SULTs. The interactions between phthalate monoesters and SULTs were analyzed, including hydrogen bonds and hydrophobic contacts.

### Statistical Analysis

Experimental data were expressed as the mean value plus standard deviation (S.D.). Statistical analysis between two groups using a two-tailed unpaired Student’s *t*-test. Multiple groups were compared using the one-way ANOVA, and *p* < 0.05 was considered to be significant.

## Results

### Preliminary Inhibition Screening of Phthalate Monoesters Towards SULT Isoforms

**Figure 2:**
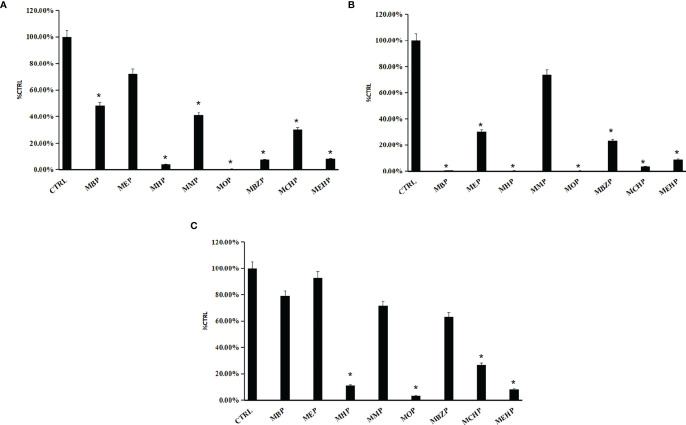
The preliminary inhibition screening of phthalate monoesters towards SULT1A1 **(A)**, SULT1B1 **(B)**, and SULT1E1 **(C)**. The data were given as mean value plus SD; *p < 0.05.

The inhibition of phthalate monoesters on SULT1A1, SULT1B1, and SULT1E1 is shown in **Figure 2**, and multiple phthalate monoesters have strong inhibition towards SULT1A1, SULT1B1, and SULT1E1. MHP, MOP, MBZP, and MEHP significantly inhibited the activity of SULT1A1. MBP, MHP, MOP, MCHP, and MEHP significantly inhibited the activity of SULT1B1. MHP, MOP, and MEHP significantly inhibited the activity of SULT1E1. As shown in **Figure 2B**, relatively more phthalate monoesters exhibited strong inhibition towards SULT1B1, with more than 80% activity inhibited by 100 μM of phthalate monoesters. All phthalate monoesters have no significant inhibition on the activity of SULT1A3 (**Supplementary Figure 1**). According to the preliminary screening results, we can find some structure–activity relationship for the inhibition of SULTs by phthalate monoesters. Phthalate monoesters with long chains inhibit the activity of SULTs relatively strongly, while phthalate monoesters with short chains (e.g., MMP, MEP, etc.) showed no significant inhibition towards all SULTs isoforms.

### Inhibition Kinetic Analysis

**Figure 3:**
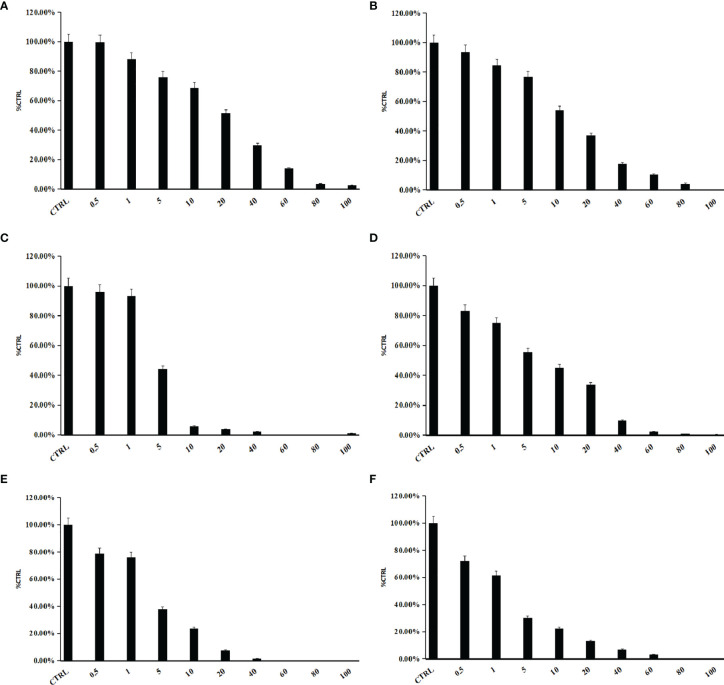
**(A)** to **(C)** present concentration-dependent inhibition of MHP towards SULT1A1 **(A)**, SULT1B1 **(B)**, and SULT1E1 **(C)**. **(D)** to **(F)** present concentration-dependent inhibition of MOP towards SULT1A1 **(D)**, SULT1B1 **(E)**, and SULT1E1 **(F)**. IC50 was determined by different concentrations of phthalate monoesters. Parallel samples were made, and the average values were used to draw the graph. Data were presented as the mean value plus SD.

**Figure 4:**
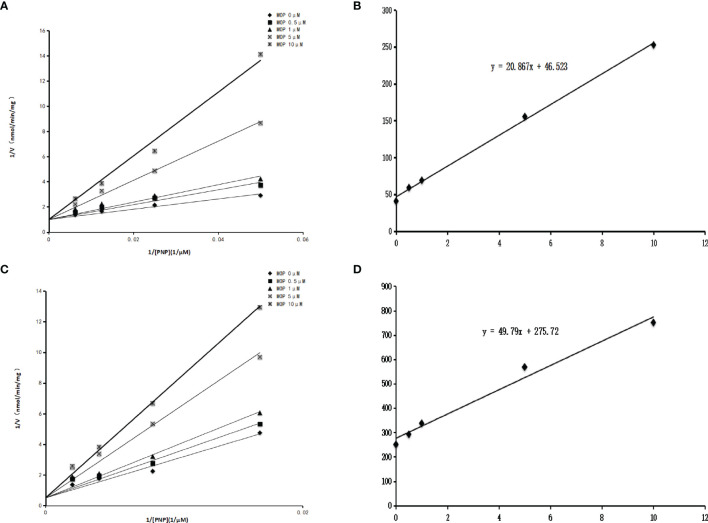
Inhibition kinetics of MOP on SULT1B1 and SULT1E1. Lineweaver–Burk plot of the inhibition of MOP on the activity of SULT1B1 **(A)** and SULT1E1 **(B)**. Each data point represents the mean value of duplicate experiments. Determination of inhibition kinetic parameter (*K*_i_) of MOP on the activity of SULT1B1 **(C)** and SULT1E1 **(D)** using the second plots. The vertical axis represents the slopes of the lines from Lineweaver–Burk plots, and the horizontal axis represents the concentrations of MOP.

Some phthalate monoesters significantly inhibited SULT1A1, SULT1B1, and SULT1E1, and IC_50_ was further determined. The concentration-dependent inhibition of MHP and MOP towards SULT1A1, SULT1B1, and SULT1E1 was exhibited (**Figure 3**). The concentration-dependent inhibition curve for other phthalate monoesters towards other SULTs isoforms are given in **Supplementary Figure 2**. The IC_50_ values for the inhibition of MHP, MOP, MBZP, and MEHP towards SULT1A1 were calculated to be 17.1, 5.6, 0.3, and 2.3 μM, respectively; IC_50_ values for the inhibition of MBP, MHP, MOP, MCHP, and MEHP towards SULT1B1 were calculated to be 8.5, 11.6, 2.7, 12.8, and 13.9 μM, respectively; IC_50_ values for the inhibition of MHP, MOP, and MEHP towards SULT1E1 were calculated to be 4.5, 1.8, and 1.7 μM, respectively (**Table 1**). In addition, inhibition kinetics were further determined, including kinetic types and parameters (*K*_i_). As shown in **Figures 4A, B**, the intersection point was located in the vertical axis in the Lineweaver–Burk plot for the inhibition of SULT1B1 and SULT1E1 by MOP, indicating the competitive inhibition of MOP towards SULT1B1 and SULT1E1. The slopes of the lines in the Lineweaver–Burk plot were calculated and drawn versus the concentrations of MOP. The second plot was used to calculate the inhibition kinetic parameters (*K*_i_). According to **Figures 4C, D**, the inhibition kinetic parameters (*K*_i_) were calculated to be 2.2 μM and 5.5 μM for the inhibition of MOP on SULT1B1 and SULT1E1, respectively.

**Table 1:** Half inhibition concentrations (IC50) of phthalate monoesters towards SULTs isoforms.

	SULT1A1	SULT1A3	SULT1B1	SULT1E1
MBP	—	—	8.49	—
MEP	—	—	—	—
MHP	17.12	—	11.57	4.54
MMP	—	—	—	—
MOP	5.61	—	2.73	1.77
MBZP	0.25	—	—	—
MCHP	—	—	12.80	—
MEHP	2.32	—	13.90	1.73

### *In Silico* Docking to Elucidate the Inhibition Mechanism

**Figure 5:**
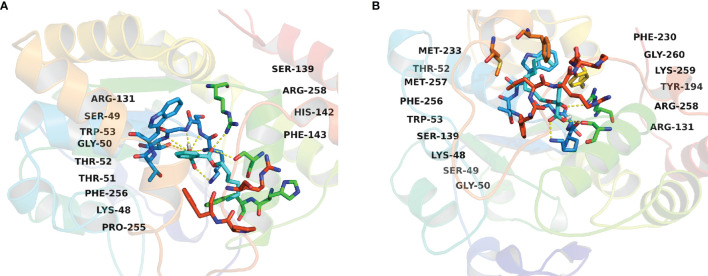
Active pocket of SULT1B1 binding with MHP **(A)** and MOP **(B)**.

**Figure 6:**
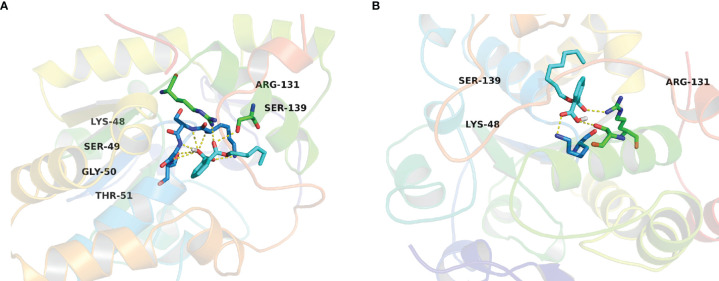
Hydrogen bonds interaction between MHP **(A)** and MOP **(B)** with the active cavity of SULT1B1.

**Figure 7:**
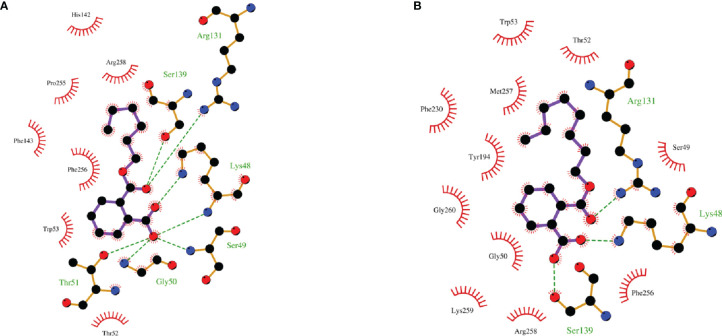
Hydrophobic interaction between MHP **(A)** and MOP **(B)** and the active cavity of SULT1B1.

Since phthalate monoesters showed broad inhibition on SULT1B1, SULT1B1 was selected as the representative SULT isoform. We used the *in silico* docking method to analyze the mechanism of its inhibition of SULT1B1. The *in silico* docking method was used to dock the chemical structure of phthalate monoesters into the activity cavities of SULT1B1, and the representative docking results of MHP and MOP were given. The active site where SULT1B1 binds to MHP consists of amino acid residues ARG-131, SER-49, TRP-53, GLY-50, THR-52, THR-51, PHE-256, LYS-48, PRO-255, SER-139, ARG-258, HIS-142, and PHE-143, as shown in **Figure 5A**. The active site where SULT1B1 binds to MOP consists of amino acid residues MET-233, THR-52, MET-257, PHE-256, TRP-53, SER-139, LYS-48, SER-49, GLY-50, PHE-230, GLY-260, LYS-259, TYR-194, ARG-258, and ARG-131, as shown in **Figure 5B**. In the binding pocket of SULT1B1, MHP formed eight hydrogen bonds to SER-139, ARG-131, LYS-48, SER-49, GLY-50, and THR-51 (**Figure 6A**), and 4 hydrophobic contacts were formed between MHP and the active cavity of SULT1B1 (**Figure 7A**). MOP formed three hydrogen bonds to SER-139, ARG-131, and LYS-48 (**Figure 6B**), and 4 hydrophobic contacts were formed between MOP and the active cavity of SULT1B1 (**Figure 7B**). The binding free energy of MBP, MHP, MOP, MCHP, and MEHP towards SULT1B1 were −7.66, −8.05, −8.23, −8.92, and −8.43 kcal/mol, respectively. The other phthalate monoester docking results are given in **Supplementary Figures 3A–C, 4A–C, and 5A–C**.

## Discussion

In this study, an *in vitro* determination system was used to investigate the inhibition behavior of phthalate monoesters on the activity of various isoforms of SULTs. The results showed that SULT1A1, SULT1B1, and SULT1E1 were strongly inhibited by some kinds of phthalate monoesters, but no significant inhibition on SULT1A3. In addition, the results also showed that short-chain phthalate monoesters, such as MMP and MEP, had no significant inhibition on all SULTs isomers, while SULT1B1 was relatively significantly inhibited.

The *IVIVE* was used to calculate the *in vivo* potential inhibition of SULTs isoforms by phthalate monoesters. MOP was chosen as the representative phthalate monoesters, and the determined *K*_i_ values were 2.23 μM and 5.54 μM for SULT1B1 and SULT1E1, respectively. According to the [I]/*K*_i_ ratio ([I]/*K*_i_ > 0.1) evaluation standard, the threshold values were calculated to be 0.223 and 0.554μM for the inhibition of MOP towards SULT1B1 and SULT1E1, respectively. Therefore, when the *in vivo* exposure concentration of MOP is greater than 0.223μM, the metabolism of endogenous substances mediated by SULT1B1 may be inhibited. When the *in vivo* exposure concentration of MOP is greater than 0.554μM, the metabolism of endogenous substances mediated by SULT1E1 may be inhibited. SULT1A1 and SULT1B1 are generally considered to be the main enzymes involved in the detoxification of exogenous drugs in the human body (18, 19), which means that phthalate monoesters may reduce the body’s detoxification of exogenous substances by inhibiting enzyme activity. SULT1E1 (estrogen sulfotransferase) is an important enzyme in hormone homeostasis regulation and biosynthesis, showing a high affinity for beta-estradiol (E2) (20), and estrogen is associated with the growth and development of human breast cancer cells (21). SULT1E1 was related to the metabolism of estrogen, so phthalate monoesters may cause certain cancer by disturbing the metabolism of estrogen. As an important phase II metabolic enzymes in human body, UDP-glucuronosyltransferases (UGTs) and SULTs play an important role in the metabolism of most endogenous substances. Previous studies have shown that UGTs can also be inhibited by phthalate monoesters (22). Therefore, because phthalate monoesters inhibit both SULTs and UGTs, it may cause greater harm to the human body.

It should be noted that phthalates undergo the first phase I biotransformation after entering the body to produce monoesters. For phthalate monoesters of different molecular weights, the way they are excreted from the body and the reactions that occur in the body may be different (7). For the excretion of phthalate monoesters, the phase II metabolic enzymes play an important role. Phthalate monoesters have stronger biological activity and toxicity, so it is of great significance to select 8 phthalate monoesters in this study to explore the inhibition of SULTs.

In conclusion, our study fully described the inhibition of phthalate monoesters towards SULT isoforms. These results will provide a new perspective for the toxicity study of phthalate monoesters.

## Data Availability Statement

The raw data supporting the conclusions of this article will be made available by the authors, without undue reservation.

## Author Contributions

J-HL and TZ designed this study. HH and B-DL performed the experiment, analyzed the data, and wrote the draft. Y-JZ, X-JF, M-CH, and G-QQ performed the instrument analysis. F-GW and YW gave critical comments and contributed to the writing of this manuscript. All authors contributed to the article and approved the submitted version.

## Funding

This research was funded by the National Key R&D Program of China (2019YFA0802300 and 2021YFA1301200), the National Natural Science Foundation of China (81970351 and 81822005), the Key Research and Development Program of Shaanxi (No. 2020SF-253), the Central University Basic Science Foundation of China (119132971000056), and the Clinical Research Award of the First Affiliated Hospital of Xi’an Jiaotong University, China (No. XJTU1AF-CRF-2017-006).

## Conflict of Interest

The authors declare that the research was conducted in the absence of any commercial or financial relationships that could be construed as a potential conflict of interest.

The handling editor [FZ] declared a shared affiliation with the author [CH] at the time of review.

## Publisher’s Note

All claims expressed in this article are solely those of the authors and do not necessarily represent those of their affiliated organizations, or those of the publisher, the editors and the reviewers. Any product that may be evaluated in this article, or claim that may be made by its manufacturer, is not guaranteed or endorsed by the publisher.
