# Supplementary material for: Inhibition of Human Sulfotransferases by Phthalate Monoesters
Source: Front Endocrinol (Lausanne). 2022 Apr 22;13:868105. doi: 10.3389/fendo.2022.868105 (PMC9072656; doi:10.3389/fendo.2022.868105)

**Supplemental Materials**

**Inhibition of human** **sulfotransferases (SULTs) by** **phthalate monoesters**

Hui Huang^1,#^, Bei-Di Lan^2,#^, Yu-Jing Zhang^1^, Xiao-Juan Fan^2^, Min-Cui Hu^3^, Guo-Qiang Qin^4^, Fei-Ge Wang^4^, Yue Wu^2^, Tao Zheng^2,*^, Jun-Hui Liu^2,5,*^

1.Department of Internal Medicine, The Affiliated Hospital of Ningxia Medical College, Yinchuan, Ningxia, 750004 P.R. China;

2. Department of CardioMetabolic Center, The First Affiliated Hospital of Xi'an Jiaotong University, Xi'an, Shannxi, 710061, P.R. China;

3. Tianjin Life Science Research Center, Department of Microbiology, School of Basic Medical Sciences, Tianjin Medical University , Tianjin , People's Republic of China;

4. Human Resources Department, The First Affiliated Hospital of Jinzhou Medical University, Jinzhou, China;

5. Department of Clinical Laboratory, The First Affiliated Hospital of Xi'an Jiaotong University, Xi'an, Shannxi, 710061, China.

# These two authors equally contributed to this work.

Corresponding author:

Tao Zheng, Department of CardioMetabolic Center, The First Affiliated Hospital of Xi'an Jiaotong University, Xi'an, Shannxi, 710061, P.R. China; Tel: +8602985323663; Fax: +8602985323715;

Jun-Hui Liu, Department of CardioMetabolic Center, The First Affiliated Hospital of Xi'an Jiaotong University, No. 277, West Yanta Road, Xi'an, Shannxi, 710061, P.R. China; Tel: +86-29-85323805; Fax: +86-29-85323729; E-mail: **liu1109@xjtu.edu.cn**;

**Supplemental** **Figure 1.** The preliminary inhibition screening of phthalate monoesters towards SULT1A3.The data were given as mean value plus S.D.,*p<0.05.


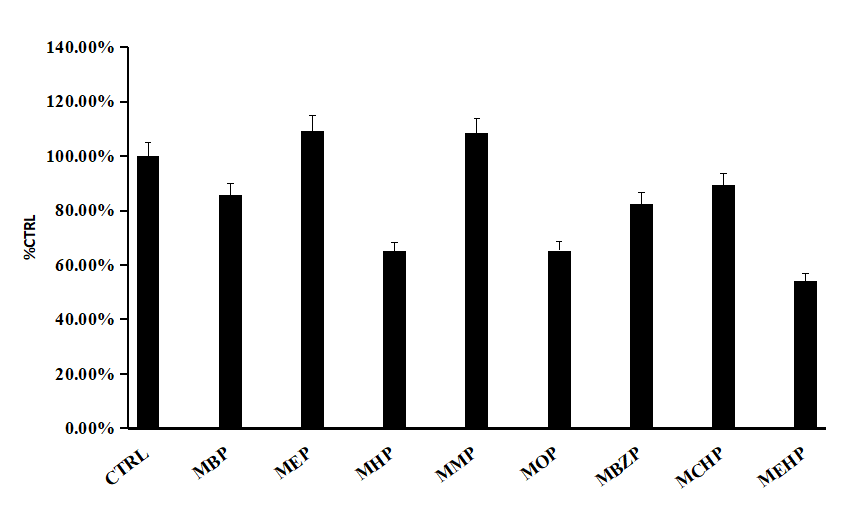


**Supplemental Figure 2.** Concentration dependent inhibition of phthalate monoesters towards SULTs.IC50 was determined by different concentrations of phthalate monoesters.Parallel samples were made, and the average values were used to draw the graph.Data were presented as the mean value plus S.D.

**
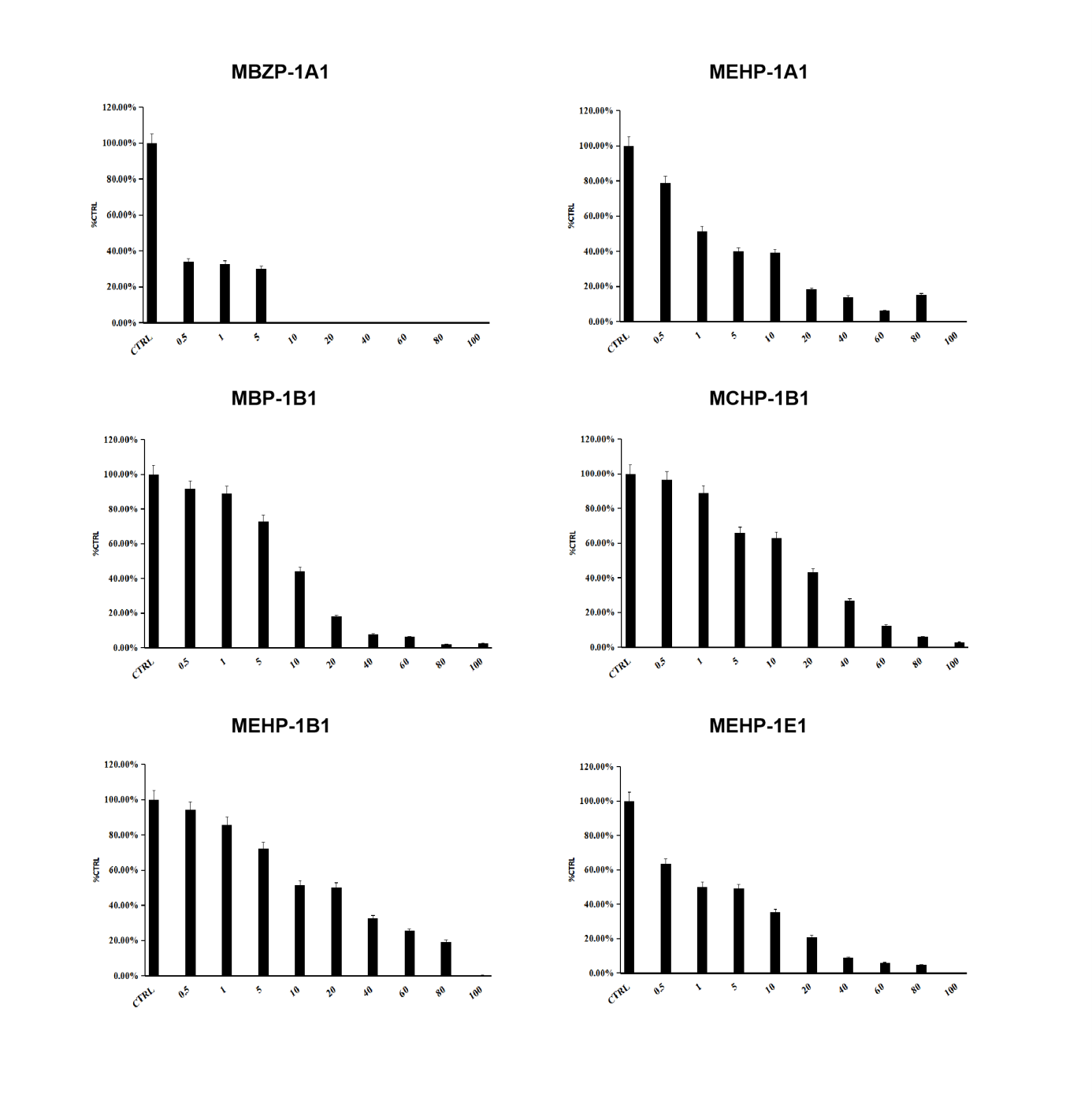
**

**Supplemental Figure 3.** Active pocket of SULT1B1 binding with MBP(A),MCHP(B)and MEHP(C).

**
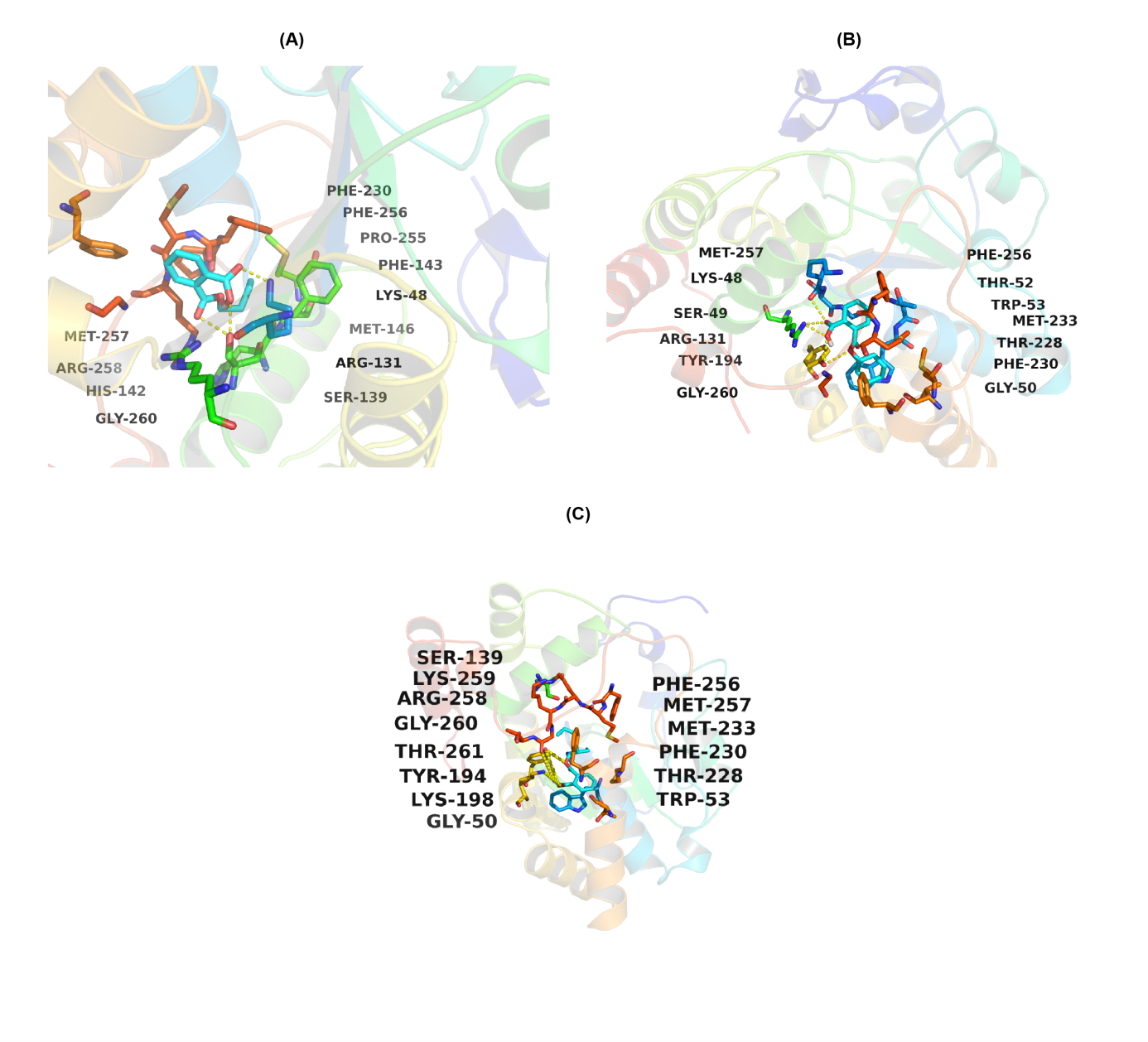
**

**Supplemental Figure 4.** MBP(A), MCHP(B) and MEHP(C) interact with the hydrogen bonding of SULT1B1 active cavity.

**
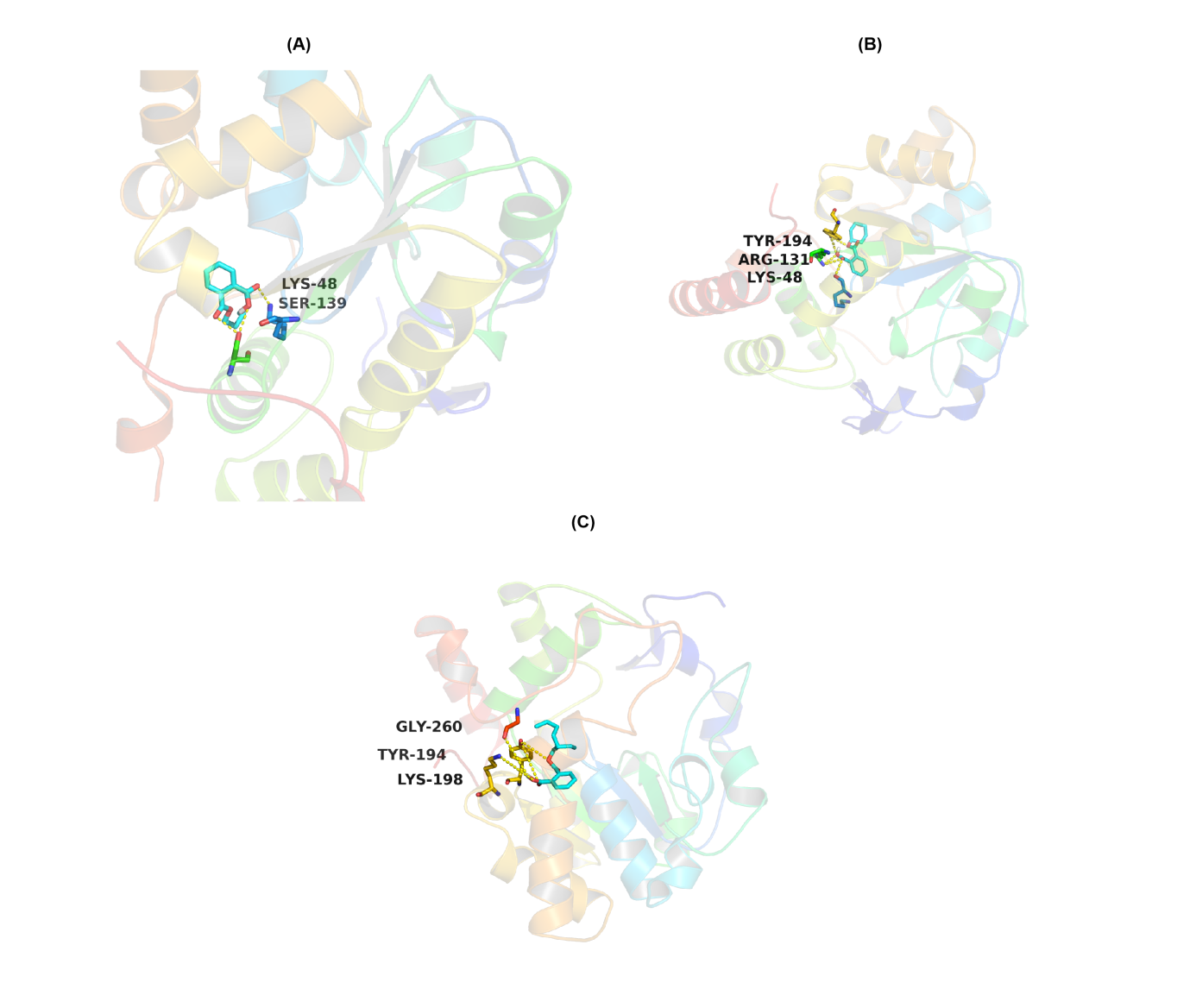
**

**Supplemental Figure 5.** The hydrophobic interaction of MBP(A), MCHP(B) and MEHP(C) with the active cavity of SULT1B1.


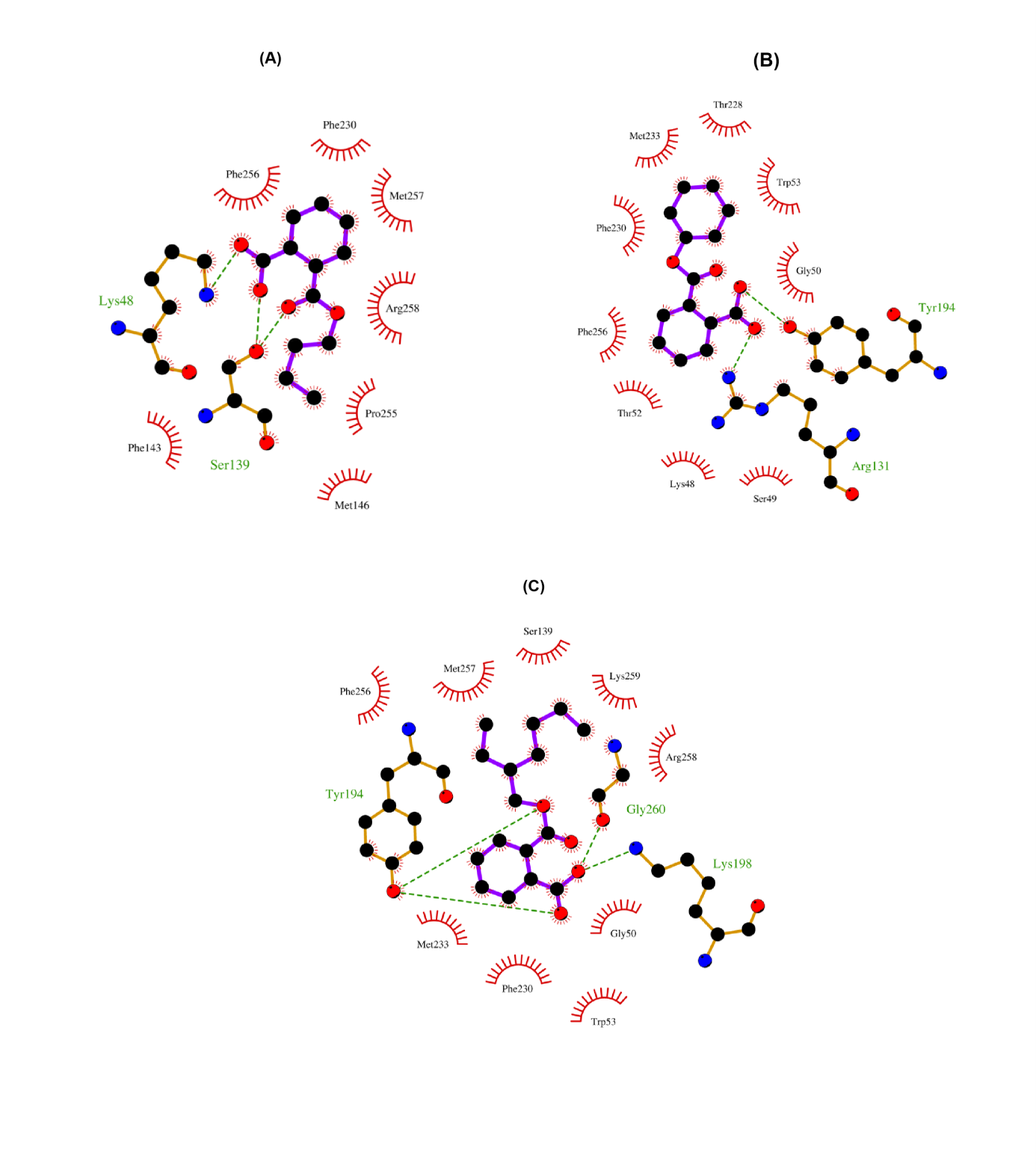

Supplement: Supplementary file 1 [file DataSheet_1.docx]
